# Transitivity in health utility measurement: An experimental analysis

**DOI:** 10.1186/2191-1991-1-12

**Published:** 2011-08-30

**Authors:** Ulrich Schmidt, Michael Stolpe

**Affiliations:** 1 Kiel Institute for the World Economy, Kiel, Germany; 2 Department of Economics, Christian-Albrechts-Universität, Kiel, Germany

**Keywords:** Transitivity, health utility, errors

## Abstract

Several experimental studies have observed substantial violations of transitivity for decisions between risky lotteries over monetary outcomes. The goal of our experiment is to test whether these violations also affect the evaluation of health states. A particular feature of our experimental design is that it takes into account the possible role of decision errors for generating violations of transitivity. Since we find neither substantial nor systematic deviations from transitive choice behaviour, we can conclude that previously reported violations do not seem to bias health utility measurement.

## 1 Introduction

Health utility measurement plays an important role in medical decision making, in particular in cost-effectiveness analyses of alternative treatments. A central assumption in health utility measurement is that preferences satisfy transitivity. Transitivity demands that whenever option A is preferred to option B and B is preferred to C, then A has to be preferred to C. In the absence of transitivity, a well-defined utility function U (i.e. for all options A and B we have U(A) ≥ U(B) if and only if A is weakly preferred to B) does not exist. Consequently, standard methods in health utility measurement, such as the time tradeoff method or quality adjusted life years, cannot be meaningfully applied in the absence of transitivity. Several empirical studies observed substantial violations of transitivity, in particular for choice between risky options (e.g. [1-8]). The validity of health utility measurement would be seriously challenged if these violations carried over to the evaluation of health states: No consistent rankings of health states could be established and meaningful outcomes measures in many applications of cost-effectiveness analysis would simply become unavailable. However, since the evaluation of health states involves consequences composed of several attributes (i.e. at least health status and life duration), behaviour may be fundamentally different than in the above mentioned studies which are based on monetary consequences.

To the best of our knowledge, it has not been analyzed before whether transitivity empirically holds for the evaluation of health states. However, several studies observed preference reversals in health utility measurement (e.g. [9-11]) which are closely related to violations of transitivity. Preference reversals are an intensively discussed phenomenon in decision making under risk. Here, a preference reversal occurs if a risky option p is preferred to another risky option q (i.e. p ≻ q) in a straight choice, but a higher certainty equivalent (CE) is assigned to q (i.e. CE(q) > CE(p)). Assume such a reversal and consider an amount z with CE(q) > z > CE(p). Since the certainty equivalent of an option should be indifferent to that option, we get q ~ CE(q) ≻ z, z ≻ CE(p) ~ p, and p ≻ q from choice. Hence, we have an intransitive cycle p ≻ q ≻ z ≻ p, i.e. an intransitive preference cycle. Precisely this result has been utilized by many studies mentioned above-in particular by [2,3,5,8]-in order to derive experimental designs from the preference reversal literature where substantial violation rates of transitivity have been observed. In one example from [3], 28 subjects exhibited the cycle p ≻ q, z ≻ p, and q ≻ z, whereas only one subject exhibited the opposite pattern-with lotteries given by the following state-dependent payoff-triples: p = (7.5, 7.5, 1), q = (10, 3, 3) and z = (5, 5, 5), where the first state has a probability of 0.4 and the second and third states a probability of 0.3 each. The motivation of our paper is that the existing evidence of preference reversals in health utility measurement suggests violations of transitivity might also occur in the evaluation of health states.

There is, however, some debate concerning the evidence of violations of transitivity [12-19]. In particular, it has been argued that the observed violations are not "true" violations since they may be simply caused by random errors. Consequently, we develop an experimental design which allows us to discriminate between true violations and violations caused by random error. Our design is presented in the next section where we also discuss the role of errors as potential causes of transitivity violations. Section 3 reports the results of our experiment and Section 4 offers some concluding observations.

## 2 Experimental Design and the Role of Errors

**Figure 1:**
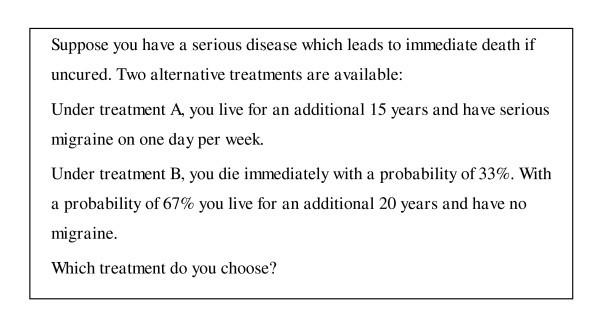
**Presentation of alternatives**.

As most other studies on transitivity, our experiment was conducted as a classroom experiment. We ran two studies with undergraduate economics students at the University of Kiel, a pretest with 40 subjects and a main test with 98 subjects. The purpose of the pretest was merely to adjust the attribute values of the alternatives for the main test so that each has a similar chance to be chosen. In both studies, subjects had to make six pairwise choices which were composed of two series with three alternatives each. Alternatives were described as in Figure 1, which presents the choice between alternatives A and B of series I.

Each alternative is characterized by three attributes: (i) the probability of immediate death, (ii) the number of additional life years in case of survival, and (iii) the number of days with migraine per weak. The corresponding values of our design are presented in Table 1. In the main test, we revised the attribute values of some alternatives (i.e. B and C in Series I and C in Series II) used in the pretest, as explained in more detail in Section 3. In these cases, the attribute values of the pretest appear in parentheses. In both tests, attribute values of the alternatives were chosen such that the majority rule implies intransitive cycles. The majority rule implies that an alternative A is preferred to another alternative B, if the number of attributes for which A is better than B exceeds the number of attributes for which A is worse, i.e.

**Table 1:** Experimental Design of Series I (left panel) and Series II (right panel)

	Prob (Death)	Years	Migraine		Prob (Death)	Years	Migraine
A	0	15	1	A	15	30	3

B	33 (35)	20	0	B	25	35	2

C	13 (10)	30	2	B	22 (20)	24 (25)	1

(1) A ≻ B ⇔ #{i: Ai ≻ Bi} > #{i: Bi ≻ Ai},

where A_i _and B_i _denote the attribute values of A and B. It is easy to see that for the values in Table 1 we have for Series I according to the majority rule B ≻ A (more years and less migraine), C ≻ B (more years and lower probability of death) and A ≻ C (lower probability of death and less migraine). Also for Series II the majority rule implies the cycle B ≻ A ≻ C ≻ B.

In the pretest, all subjects received an identical booklet with all six choices, each choice on a separate sheet. In the main test there were two booklets, one with three choices (two from one series and one from the other series) at the beginning of class and a second booklet with the remaining three choices at the end of class. This procedure ruled out that subjects could make consistency checks. The main test also divided subjects into two groups, to control for ordering effects. Compared to the first group, the order of the two booklets and the order of alternatives in the booklets were reversed in the second group. Since there are no significant differences in the results for these two groups we do not distinguish them in the following. Finally, in order to motivate subjects for careful consideration of the questions, 12 subjects drawn randomly received a flat payment of 20 Euros in the main test.

In the studies of [2,3,5,8], intransitivities are tested by the so-called cycling asymmetry. With three choices, there exist two possible intransitive response cycles and the studies conclude that observed intransitivities are not simply noise if one cycle occurs with a significantly higher frequency than the other. The validity of this argument has been questioned by [14]. Consider for instance the result for Case 2 of [5] presented in Table 2. The entries in the first row give the possible choice patterns with which a subject could respond to the three questions and the second row gives the number of subjects (out of a total of 90 subjects) who have actually responded with a given pattern. For example ABA means that a subject chose A over B in the first choice, B over C in the second one, and A over C in the third one. Altogether, there are eight different possible response patterns out of which the last two (ABC and BCA) are intransitive. Suppose that all subjects have transitive preferences but they sometimes make errors when choosing between lotteries. It is easy to see that the cycle ABC can be caused by only one error if the true preferences are ABA, BBC, or ACC. Analogously, the cycle BCA is caused by one error if the true preferences are ACA, BBA, or BCC. According to Table 2, 18 subjects chose patterns ABA, BBC, or ACC while 69 subjects chose ACA, BBA, or BCC, i.e. the latter three patterns occur much more frequently. As the simplest case we can assume that the probability of errors is the same for all subjects and independent of the underlying true preference pattern. This implies that errors will lead much more frequently to the cycle BCA than to the cycle ABC given that the true preferences are always transitive. The observed differences in those frequencies thus may not be taken as evidence of intransitivities.

**Table 2:** Results of Starmer and Sugden (1998) [5]

	ABA	ACA	BBA	BBC	ACC	BCC	ABC	BCA
Case 2	5	2	12	9	4	45	3 (3.3%)	10 (11.1%)

To control for the possible role of errors, [18,19] estimate an explicit error model using data from an identically repeated experiment. However, this procedure requires additional assumptions on the structure of the error model. Moreover, it is unclear whether the error model does accommodate real behaviour sufficiently well, and if not, it is hard to interpret the results. We therefore follow a different route in this article: We simply adjust the attributes of the alternatives such that the number of subjects choosing patterns ABA, BBC, or ACC roughly equals the number of subjects choosing the other three transitive patterns ACA, BBA, or BCC. In this case random errors would imply an approximately equal frequency of the two intransitive patterns so that we can use the cycling asymmetry to test for intransitivities. Given the categorical nature of our observations, we shall use a one-tailed binomial test to check if the null of equal frequency is rejected in favour of a greater frequency of one intransitive pattern in line with the cycling asymmetry.

## 3 Results

### (i) the pretest

Table 3 presents the results of the pretest. Again, the first row of the table gives the possible response patterns while the second row gives for each pattern the number of subjects who responded with this pattern. The table shows that there are no intransitivities for Series II whereas 10% of subjects responded with intransitive cycles for Series I; yet both cycles occur with equal frequency and no cycling asymmetry can be observed. However, the main goal of the pretest was not to test transitivity but to help adjust the alternatives for the main test as outlined in the preceding section. The frequency of patterns which can lead to the cycle ABC by one error (ABA, ACC, and BBC) is 16 for Series I and 9 for Series II. The frequency of the opposite patterns (BCC, BBA, and ACA) is given by 20 for Series I and 31 for Series II. These observations show that in particular Series II was unbalanced in the pretest, mainly due to the fact that alternative C is preferred too frequently. We therefore made the attributes of this alternative less attractive in the main test. For Series I, we also made alternative B slightly more attractive as indicated in Table 1.

**Table 3:** Results of the Pretest

	ABA	ACA	BBA	BBC	ACC	BCC	ABC	BCA
Series I	0	11	5	2	14	4	2 (5.0%)	2 (5.0%)

Series II	2	8	2	5	2	21	0	0

### (ii) the main test

The results of the main test are presented in Table 4. We can verify that our alternatives are rather balanced as the frequency of patterns ABA, BBC, and ACC (52 for Series I and 47 for Series II) is relatively close to the frequency of patterns BCC, BBA, and ACA (40 for Series I and 43 for Series II). The incidence of intransitivities is rather limited compared to previous studies which reported substantial violations of transitivity. In our study only 6.1% of subjects violated transitivity in Series I and 8.1% of subjects in Series II.

**Table 4:** Results of the Main Test

	ABA	ACA	BBA	BBC	ACC	BCC	ABC	BCA
Series I	9	15	12	14	29	13	5 (5.1%)	1 (1.0%)

Series II	20	6	7	20	7	30	6 (6.1%)	2 (2.0%)

In both series, the cycle ABC occurs more frequently than the cycle BCA. However, according to a one-tailed binominal test (which was also used by [5] and is appropriate for observations falling into two categories, such as ours, where only a *greater *sample frequency of observations in one category than expected under the null hypothesis justifies rejection), this difference is not statistically significant, so we can not conclude that a cycling asymmetry exists. Since the rarely observed cycle BCA is the one implied by the majority rule for our design, this rule appears to perform rather poorly in our experiment.

## 4 Conclusions

This paper has presented an experiment aimed at testing transitivity in the valuation of health states, as required in health utility assessments. A particular feature of our design is the use of a balanced set of alternatives such that an asymmetric frequency of the two intransitive cycles is unlikely to result from random errors. In contrast to many previous experimental studies, we find neither a substantial frequency of subjects violating transitivity nor a significant cycling asymmetry. Further research may be required to understand whether the absence of the intransitivity problem is mainly due to the control for errors in our design or due to the fact that our alternatives are not lotteries over monetary amounts, as in previous studies, but composed of health states. Moreover, additional studies of transitivity in the health domain would be useful trying to overcome some of the obvious limitations of our study which include a non-representative sample of students and the small number of stimuli. In particular, the fact that many of our subjects had no experience with the presented health states (i.e. days per week with migraine) may have contributed to our results.

Altogether, we do not find evidence that people's evaluation of discrete health states is substantially biased by violations of transitivity. This finding is in line with other recent experimental studies [18-20] that test transitivity while controlling for the possible role of errors. If transitivity holds, the evaluation of any one alternative does not depend on the other alternative with which it is compared. This is a necessary prerequisite for many tools and concepts in the health domain like quality-adjusted life years or cost-effectiveness analyses. It is therefore encouraging that there really does not seem to be much evidence for intransitive choice being a reality.

## Competing interests

The authors declare that they have no competing interests.
